# Female vs. Male Ampelmännchen-Gender-Specific Reaction Times to Male and Female Traffic Light Figures

**DOI:** 10.3389/fpsyg.2017.00690

**Published:** 2017-05-23

**Authors:** Farid I. Kandil, Bettina Olk, Claus C. Hilgetag

**Affiliations:** ^1^Department of Computational Neuroscience, University Medical Center Hamburg EppendorfHamburg, Germany; ^2^Department of Psychology and Methods, Jacobs University BremenBremen, Germany; ^3^Applied Psychology, HSD University of Applied SciencesCologne, Germany; ^4^Health Sciences, Boston UniversityBoston, MA, USA

**Keywords:** perceptual conflict, reaction time, stroop effect, gender effect, traffic, pedestrians, traffic safety

## Abstract

Traffic signs are important visual guiding signals for the safe navigation through complex road traffic. Interestingly, there is little variation in the traffic signs for cars around the world. However, remarkable variation exists for pedestrian traffic signs. Following up from an earlier study, we investigated the visual efficacy of female vs. male German Ampelmännchen pedestrian traffic signs. In a Stroop-like test, 30 subjects were presented with female and male go and no-go traffic light figures that were shown either in the corresponding or opposing color. Subjects had to indicate, based either solely on the form or the color of the figure, whether they were allowed to go. Accuracy and response times across all subjects did not differ for the female vs. male signs, indicating that Ampelfrau and Ampelmann signs have equal visual efficacy. However, subjects responded faster to signs of their own vs. the opposite gender. This preference for signs of one's own gender is in accordance with effects in social psychology described by social learning theory. An introduction of such novel traffic lights may, thus, contribute to higher compliance with the traffic sign signals.

## 1. Introduction

When navigating busy road traffic, pedestrians, cyclists, and car drivers are challenged by multitudes of multi-sensory information. In order to navigate safely, traffic participants need to focus on relevant aspects of the situation, such as cars and bicycles that may slow down or change lanes and pedestrians who cross roads unpredictably, and follow the instructions of street and traffic signs. At the same time, they must disregard irrelevant distractors, such as shops and advertising displays. During the last decades, the traffic situation in cities has become more and more complex. With ever increasing traffic and a larger number of colorful and animated advertisement in shops and on billboards, the number of distractors has increased. Thus, the need to select the important pieces of information from the wealth of visual information puts an ever higher workload on our visual system.

**Figure 1:**
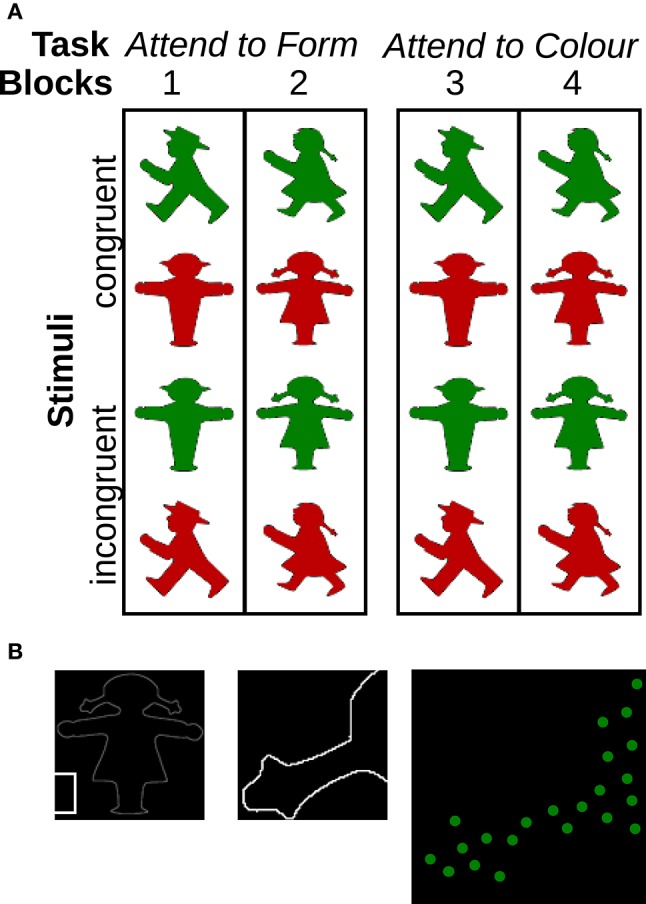
**Experimental design and construction of the stimulus. (A)** In this color-form Stroop-like reaction time task, subjects had to decide whether they could go or had to stop. They were instructed to attend to the target cue, i.e., the color cue in blocks 1 and 2 and the form cue in blocks 3 and 4, and to neglect the other cue altogether. The to-be-neglected cue could be either congruent (upper two rows in the panel) or incongruent (lower two rows) with the target cue. East German male and female traffic light figures were tested. **(B)** To keep the total amount of light energy constant across the figures, which covered varying amounts of area, we did not present the figures as such but only a fixed number of randomly placed virtual (red or green) pixels within the outline of each figure.

More than 50 years ago, in 1961, German traffic psychologist Karl Peglau noticed the increasing degree of traffic, the rising number of traffic accidents and the associated death toll at that time. In order to reduce confusion arising from shared signs for drivers and pedestrians, Peglau suggested installing specific traffic lights for each kind of road user. Given that pedestrians are the most vulnerable group, he stressed the importance of developing particularly appealing, instructive and clearly distinguishable go and stop signs for pedestrians (Brieler, 2010; Ampelmann, 2016c). Beforehand, traffic lights for pedestrians showed either a flashing pedestrian figure on top and the words for “wait,” “attention,” and “go” beneath it, or simply used a smaller version of the red-yellow-green circular signals that are also used for car drivers. The stylized signs that Peglau invented, and which then became the official traffic light figures in the former East Germany around 1970, show a man either standing still with his arms stretched out in red (stop sign) or the same man, but in a wide-paced walk in green (go sign) as illustrated in the upper two figures in the left column of Figure 1A. The combination of both features, form and color, was not only intended to facilitate and improve the visual and cognitive perception of the signals, but also to increase the intuitive appeal of the signals to pedestrians (Brieler, 2010; Ampelmann, 2016c).

At the same time, in West Germany, a more neutral pair of traffic light figures was introduced, which look similar to most other European traffic light figures. The main contrast to the East German figures is that the West German figures are more abstract, without detailed features, such as a hat or shoes and that the stop figure simply stands still with the arms by its side.

After German unification in 1990, the two traffic light figures triggered a debate of the relative superiority of the East or West German “Ampelmännchen.” Specifically, the old-fashioned appearance of the East German figure with its hat was claimed to possess high visual efficacy (Ampelmann, 2016a). The figure has become an object of affection and “ostalgia” — a pun derived from “ost,” the German word for East, and “nostalgia.” Duckenfield, Calhoun and Moran noted that “East German opposition to the relentless Westernization […] was articulated in a well-publicized campaign to save the cute, jauntily-hatted “little lamp man” on GDR traffic lights from being replaced by his characterless West German counterpart” (Duckenfield and Calhoun, 1997; Moran, 2004). Eventually, the general replacement of East German Ampelmännchen signs by West German signs was stopped, and in more recent years the East version has also been introduced in some West German cities (Dobrinkat and Brunner, 2005).

As a side-effect of this movement, in 2004 a female version of the East German traffic light man, a so-called “Ampelfrau,” was designed (Reuters, 2012; Ampelmann, 2016b) and installed at some crossings in the East German cities of Zwickau and Dresden, and in 2010 also at one crossing in the West German city of Cologne (Ampelmann, 2016b).

In a previous project, we investigated the relative visual efficacy of the (male) East and West traffic light figures (Peschke et al., 2013). To this end, we used a derivative of the Stroop test, one of the most prominent paradigms to investigate the control of attention (Stroop, 1935). This paradigm requires the suppression of involuntary processing of task-irrelevant attributes of a stimulus in favor of paying attention to less automatically processed task-relevant attributes (MacLeod, 1991). These demands make the Stroop task very informative with respect to the examination of attentional control (Banich et al., 2000). In the classic Stroop paradigm, subjects are presented with color words printed in either the same color as indicated by the word (congruent condition), for example, the word “red” printed in red, or words printed in a differing color (incongruent condition). Subjects are then asked to name the print color, and in some studies also to read out the names as a means of control. In these cases, responses typically are faster and more accurate in the congruent than in the incongruent condition. Moreover, in the two conflicting incongruent conditions, the one in which subjects have to read the written word typically produces smaller reaction times (and higher accuracy) than the one in which subjects are instructed to name the print color. Thus, word reading is more robust and less prone to conflicting information than color naming. This finding is traditionally interpreted to mean that word reading is highly automatized (Fraisse, 1969).

In our previous study (Peschke et al., 2013), we used a Stroop-like paradigm to test the efficacy of East vs. West German traffic light figures. Specifically, we measured accuracy and reaction time for each figure in their normal (i.e., congruent) color, that is, the walking figure was shown in green and the standing figure was shown in red, and compared performance for East and West German figures in order to determine whether participants respond faster and more accurately to one or the other. Moreover, we tested the robustness of the figures against conflicting (i.e., incongruent) information.

While reaction times for both variants were similar, we found that robustness of the visual perception against conflicting Stroop information was higher for the East German signs (Peschke et al., 2013). The findings suggested that simple measures of accuracy, reaction times, and robustness against distracting information can be used to assess the efficacy of traffic signals and thus serve to increase road safety.

In the present project, we used the same Stroop-like approach to test the efficacy of (East German) male and female traffic light figures. Furthermore, we asked whether there exist gender-specific interactions between pedestrians and traffic light figures of both sexes. In particular, there are four conceivable hypotheses: (i) a main effect for gender in favor of the male version of the sign because most subjects are used to encountering male traffic light figures (adaptation); (ii) a main effect for gender in favor of the female figure, as it is new and may raise more attention (novelty); (iii) an interaction between gender of the figure and gender of the subjects in the way that female subjects attend better to male traffic light figure and vice versa (attraction), and finally (iv) female and male subjects may respond better to instructions given by traffic light figures of their own sex (identification).

There is much support for the identification hypothesis (iv) derived from social psychology, and in particular from social learning theory (e.g., Bandura, 1977). The theory posits that most human behavior is learned by observing and imitating other people, so called role models. People learn the easier the more they identify themselves with the role models, as has been demonstrated in a number of experiments during the last decades. Children learned aggressive behavior better from a role model shown in a film if they had the same gender (Bandura et al., 1963). Children also preferred same-sex role models when these engaged in social behavior (Slaby and Frey, 1975) and even in unpleasant duties (Perloff, 1982). School students identified with singers of the same rather than opposite gender (Killian, 1990) and young adults identified more with same-sex professors (Gilbert et al., 1983). These findings from social psychology are corroborated by a recent neuroscientific study (Losin et al., 2012) demonstrating that imitation of own-gender models as opposed to other-gender models activates the striatum, an area associated with classical reward tasks.

Here, we presented traffic light figures of two variants (male and female) in congruent colors (red stop signals and green go signals) or incongruent colors (green stop signals and red go signals). By instructing subjects to decide whether to go or to stop based on either the color (blocks 1+2) or the form (blocks 3+4) of the figure (cf. Figure 1A), we measured robustness of the form/color perception under distracting conditions. From our previous results (Peschke et al., 2013) and neuropsychological findings (Bach and Meigen, 1998; Regan, 2000; Fahle et al., 2003; Kandil and Fahle, 2003), it was expected that color-based decisions are reached faster, with higher accuracy and higher robustness. Thus, like in the classical Stroop test, the main focus of this project was on comparing the measures for form perception between the male and female figures and their interaction with the gender of the subjects.

## 2. Methods

### 2.1. Subjects

A total of 30 subjects, between 19 and 26 years of age, 15 male and 15 female, participated in this study. They were pre- and post-graduate students recruited at Jacobs University Bremen and at University Medical Center Hamburg Eppendorf. Subjects reported absence of a neurological or psychiatric history, and abstinence from alcohol during 24 h, and any other drugs (except nicotine) during at least 1 week prior to the testing. All subjects were naïve to the purpose of the study, and had normal or corrected to normal visual acuity. Subjects received either course credits or a chocolate bar for their 10-min participation. Experimental data from all subjects were obtained, stored and analyzed fully anonymized. No subject stemmed from, or had lived in, Berlin or the five East German States. Thus, the subjects' experiences with these traffic light figures were (at the point of testing) restricted to occasional visits to Berlin or other East German cities. This study was carried out in accordance with the recommendations of the German Psychological Society/Deutsche Gesellschaft für Psychologie (DGPs) with written informed consent from all subjects. All subjects gave written informed consent in accordance with the Declaration of Helsinki. The protocol was approved by the ethics' committee of the German Psychological Society (DGPs).

### 2.2. Design

The experiment consisted of a color-form Stroop task applied in four blocks (Figure 1A). Subjects were shown pictures of traffic-light signals either in congruent colors, with the “go” signal in green and “stop” signal in red, or in incongruent colors, that is a “go” signal in red and “stop” signal in green. Their task was to respond as fast and as accurately as possible. In blocks 1 and 2, subjects were instructed to make “go” or “stop” responses based on the shape of the figure only, whereas in blocks 3 and 4, they had to base their response on the color information of the stimulus only. Two different traffic light figures were tested, the East German male traffic figure and its female counterpart (see “Stimuli” section for details). Within each block, only one figure type was presented, so blocks 1 and 3 showed the male and blocks 2 and 4 the female pedestrian signs. The order of the figures (male first vs. female figures first) and the tasks (color first vs. form first) were balanced across subjects. In each block, after a practice period of 5 trials, 80 trials were presented in random order, that is, 20 trials for each of the four conditions: (1) “go” signal in green (i.e., congruent condition); (2) “go” signal in red (incongruent condition); (3) “stop” signal in green (incongruent), and (4) “stop” signal in red (congruent). Each trial started with the presentation of a central fixation cross for 750 ms (from 1,000 to 250 ms prior to stimulus onset). The stimulus itself was displayed centrally on the screen. Presentation ended either with the response or after 5,000 ms, whichever came first. Subjects responded by pressing one of two gray buttons on a response box labeled “GO” and “STOP” using the index or middle finger of their dominant hand. The presentation of the stimulus was followed by a blank screen, presented for a random period between 1,000 and 2,500 ms, to prevent subjects from following a monotonous temporal response pattern.

### 2.3. Stimuli

Female and male East German traffic light figures (“TLFs”) were photographed by the first author on site in Berlin and Brandenburg, Germany. Photographed figures were corrected for different camera angles and then converted into black (background) and white (figure) images of approximately the same height. All figures covered an area of 34.5–36.5% of the surrounding square and were, thus, approximately equal in size. However, in order to rule out even spurious size effects, we did not use the images *per se* but construed derivatives (Figure 1B, from left to right). For each of the original figures, 500 small dots were randomly positioned within the figure outline. This approach ensured that the intensity of visual stimulation was the same across all four figures. Full figures subtended an area of 16 × 16 cm on the screen, corresponding to 16 × 16° of visual angle when viewed from a distance of 57 cm. Each of the 500 single dots presented within the outline of the figure consisted of a Gaussian spot and had a width of 4 pixels (1.2 mm). In total, dots covered approximately 1/12 of the figure area. Subjects reported that the stimuli looked similar to modern traffic lights in which figures are made up of individual LED diodes. Dots appeared either as bright red or bright green dots (20.0 cd/m^2^) against a dark background (0.1 cd/m^2^), with a high Michelson contrast of 99%. Isoluminance between red and green dots was set using a monitor calibration device (Spyder Elite 4, Datacolor Inc., Lawrenceville, NJ, USA), and confirmed by a photometer (Gossen GmbH, Nürnberg, Germany). Isoluminant stimuli were used here to prevent subjects from deriving their answers based on luminance rather than color information, because luminance information is known to be processed faster in the visual system (Bach and Meigen, 1998; Regan, 2000; Fahle et al., 2003; Kandil and Fahle, 2003).

### 2.4. Presentation

Stimuli were presented on a 27-inch back-lit TFT monitor (iiyama G2773HS) with a fast response time of nominally 1 ms and a vertical refresh rate of 120 Hz, using a standard Linux-PC (Ubuntu 14.04 LTS) with a dedicated graphics board (nVidia GeForce GTX 750). Stimulus presentation was controlled by a custom-written C-program, which also acquired responses from a custom-built response box that was monitored via the parallel port with a temporal resolution of 1,000 Hz. The synchrony between all temporally critical parameters, such as the refresh rate of the monitor, the response time of the box and the uptake-time of the program were controlled and verified using trigger signals, photo-sensitive LEDs and a clock-pulse generator in conjunction with an oscilloscope.

Subjects were seated in a dimly lit room and viewed the stimuli from a distance of 57 cm.

### 2.5. Data processing

Preprocessing of the data was performed separately for each subject and each block of the experiment. We considered three aspects of the responses: (i) Accuracy was established as the percentage of correctly answered trials in each block and subject. Accuracy was used to compute the (ii) corrected Reaction Times (cRT). Following Rach et al. (2010), reaction times were corrected by dividing the reaction time for every single trial *j* by the accuracy in that block, hence:

(1) $$cRT_{j}=RT_{j}/accuracy$$

Thus, reaction times were increased for every incorrectly answered trial. These corrected reaction times were then pooled across the two *congruent* conditions (go signal in green and stop signal in red) and the two *incongruent* conditions (go signal in red and stop signal in green), and averaged using the median. In total, this approach gave eight resulting average cRTs per subject: 2 tasks (color vs. form) × 2 stimuli (male vs. female TLFs) × 2 levels of congruency (congruent vs. incongruent). (iii) Stroop Effects. The difference between the corrected reaction times for the congruent and the incongruent conditions in each of the (2 tasks × 2 stimuli =) 4 blocks represented the Stroop effect:

(2) $$Stroop\;Effect={\bar{cRT}}_{incongruent}-{\bar{cRT}}_{congruent}$$

Thus, if subjects required more time to respond correctly to an incongruent than a congruent stimulus, this resulted in a Stroop effect.

### 2.6. Data analysis

Group data were analyzed using non-parametric tests. While the number of subjects per group (*n* = 15) was large enough to allow analysis with parametric tests, reaction times can neither be expected to be normally distributed nor to be symmetrical (Ratcliff, 1993). This applies to both, RTs across the trials of a given condition and the averaged RTs across the subjects of a group (Ratcliff, 1993; Van Zandt and Townsend, 2013). This problem can be overcome in three ways: using mean, standard deviation and parametric methods with either (i) the inverse RTs (i.e., 1/RT), or (ii) the logarithm of the RTs (i.e., log(RT)), or (iii) by using more robust statistical approached, such as the median RT and non-parametric tests. Of these alternatives, we chose the third one as non-parametric methods are more robust and avoid transformation of the RT data into arbitrary unit space.

In detail, data for the color vs. form task were compared using Wilcoxon's signed-rank test (Wilcoxon, 1945). Furthermore, data for the higher-order designs were analyzed using the non-parametric rank-based test for factorial designs, with one between-group variable and two within-group variables, devised by Brunner et al. (2002), namely the “F1-LD-F2” test. To allow for comparison to other studies in the field, we also analyzed the data using an ANOVA with repeated measures in the second and third factor. In the results section and in Tables 1–**3**, we report outcomes for both approaches. Since both result in *F*-statistics and *p*-values, we indicated the results by “Rank Test” and “ANOVA,” respectively. Decisions with respect to the significance of main effects and interactions, however, were based solely on the non-parametric rank tests. Data were analyzed using the software package R (version 3.0.2), extended by a custom-written procedure for the Brunner and Langer test. Descriptive Statistics are provided as parametric mean (M), standard deviation (SD), standard error (SE) as well as median (Mdn), and the median absolute deviation (MAD). The MAD is defined as the median of the absolute values of the deviations of the data from their median. It is more robust against outliers than the SD and the Inter-Quartile Range (Leys et al., 2013).

**Table 1:** **Descriptive statistics for accuracy and reaction times**.

**Subject G**	**Stimulus G**	**Congruency**	**Accuracy**					**Reaction times**				
			**Mdn**	**MAD**	***M***	***SD***	***SE***	**Mdn**	**MAD**	***M***	***SD***	***SE***
F	M	con	96.9	1.5	95.6	4.1	1.0	469.2	42.2	475.7	65.4	16.8
F	M	incon	96.6	1.6	94.0	4.5	1.1	493.4	35.1	498.4	61.5	15.9
F	F	con	96.9	1.5	95.9	2.9	0.7	437.8	31.5	446.6	46.4	11.9
F	F	incon	94.5	3.7	92.4	9.2	2.3	452.6	52.2	469.7	59.6	15.3
M	M	con	98.4	1.5	96.6	3.6	0.9	424.5	25.4	431.9	42.6	11.0
M	M	incon	92.9	1.6	92.1	4.4	1.1	466.0	25.5	471.9	51.3	13.2
M	F	con	96.9	1.4	95.7	3.0	0.7	450.9	20.0	452.5	52.7	13.6
M	F	incon	91.3	1.8	91.4	4.2	1.1	474.5	27.2	490.0	72.8	18.8

*Mdn, median; MAD, median absolute distance; M, mean; SD, standard deviation; number and SE, standard error; F, female; M, male; con, congruent; incon, incongruent*.

## 3. Results

Following Fidell and Tabachnick (2003), we examined the raw data with respect to their quality prior to the analysis and had to exclude two female subjects of the original group. Raw data for the first subject showed far outlying response times of RT > 700 ms, which is 5 × SD higher than the group mean. Thus, we could not assume that the subject had responded as fast as possible, but taken time to decide. In the worst case, this would have resulted also in higher Stroop effects and, thus, an overestimation of the group mean in favor of the alternative hypothesis. The second subject had a low overall accuracy of only < 75% (≈ M − 8 × SD). Closer inspection revealed that she had apparently ignored the different tasks and responded according to the color task throughout the whole experiment. We replaced both subjects by two new female subjects of the same age. All 30 subjects in the current pool responded with a high overall accuracy of 95.1% (*SD* = 3.1) and mean RTs of 435 ms (*SD* = 41.3). The accuracy, corrected reaction times, and the Stroop effect, that is, the difference between reaction times for congruent and incongruent trials, were computed separately for each block and used to test the postulated hypotheses.

### 3.1. Color vs. form task

Overall, performance was significantly better in the two blocks of the color task than in the corresponding two blocks of the form task: accuracy was higher (96.0%, *SD* = 3.46 vs. 94.4%, *SD* = 3.81, Wilcoxon's signed-rank test: *T* = 85.5, *z* = 3.02, *p* = 0.0012), overall RTs were lower (404.3 ms, *SD* = 46.84 vs. 465.6 ms, *SD* = 48.13, Wilcoxon's signed-rank test: *T* = 27, *z* = 4.23, *p* < 0.0001), and the Stroop Effect was smaller (2.21, *SD* = 28.297 vs. 30.80, *SD* = 24.61, Wilcoxon's signed-rank test: *T* = 48, *Z* = 3.79, *p* < 0.0001) in the color task than in the form task. These findings indicate that the color task was easier and less error prone for the subjects. However, since the color task had only been introduced as a control condition, all remaining analyzes were confined to the results of the form task.

### 3.2. Subject gender, stimulus gender, and congruency

A three-way non-parametric rank test (Brunner et al., 2002), as well as a repeated-measures ANOVA, were performed for the between-subjects factor “Subject Gender” and the two within-subjects factors “traffic light figure gender” (“TLF Gender”) and “Congruency.” Tests were performed separately for accuracy and reaction times. A Stroop effect would be indicated by a significant main effect of the factor “Congruency” with RT for congruent stimuli being shorter than those for incongruent ones. Furthermore, a factor would be considered to be a modulator if the interaction of that factor with the “Congruency” became significant.

Results for Accuracy (Table 2) revealed a Stroop effect, that is, a significant main effect for “Congruency” (Rank test: *t* = 66.1, *p* < 0.0001 < alpha^*^ = 0.0073; ANOVA: *F*_(1, 28)_ = 27.7, *p* < 0.0001), indicating that accuracy was higher for the congruent stimuli (Mdn = 96.9%, MAD = 1.54; *M* = 96.0%, *SD* = 3.39) than for the incongruent stimuli (Mdn = 93.3%, MAD = 3.3; *M* = 92.5%, *SD* = 5.93). Next to that, the first-order interaction between “Congruency” and “Subject Gender” became significant (Rank test: *t* = 8.8, *p* < 0.0031 < alpha^*^ = 0.0073; ANOVA: *F*_(1, 28)_ = 2.03, *p* = 0.1652), indicating a larger congruency effect in male than in female subjects (Table 1).

**Table 2:** **Inference statistics for the accuracy**.

**Accuracy**	**Rank test**			**Anova**		
	***t***	***df***	***p***	***F***	***df***	***p***
Subject G	0.767	1	0.3811	0.1420	1,28	0.7092
Stimulus G	0.997	1	0.3180	2.2734	1,28	0.1428
Cong	66.140	1	<0.0001^*^	27.7433	1,28	0.0000
Subject G × Stimulus G	1.057	1	0.3039	0.0101	1,28	0.9207
Subject G × Cong	8.758	1	0.0031^*^	2.0303	1,28	0.1652
Stimulus G × Cong	0.618	1	0.4317	0.3633	1,28	0.5515
Subject G × Stimulus G × Cong	0.003	1	0.9565	0.6145	1,28	0.4397

Test results are shown for Brunner and Langer's (non-parametric) rank-based f1.LD.f2 test and the (parametric) repeated-measures ANOVA. Significant factors are indicated by an asterisk (^*^). Assessment whether a factor became significant or not, solely relies on the non-paramtric test. The adjusted apha for 7 tests is alpha^*^ = 1−(1−0.05)^1/7^ = 0.0073. G, Gender; Cong, Congruency.

**Figure 2:**
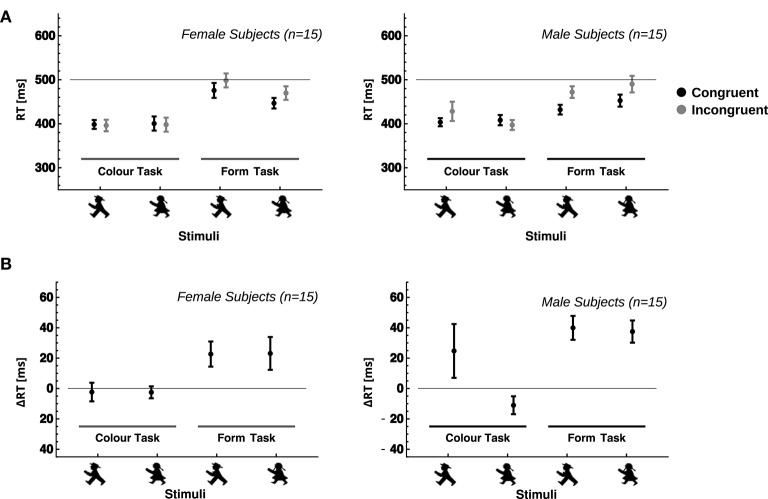
**Group results for female and male subjects**. Panels show average reaction times **(A)** and average Stroop effects **(B)** for female (left) and male (right) subjects. Dots and antennas indicate means and confidence intervals for the fifteen subjects in each gender group. Notably, the influence of the form as a distractor in the color task, which could be seen in single subjects, levels out across subjects of both genders, resulting in only insignificant Stroop effects. In contrast, there is a significant influence of color as a distractor in the blocks in which subjects were instructed to respond to the form.

Results for Reaction Times (Figure 2A and Table 3) showed a significant main effect for “Congruency” as well (Rank test: *t* = 34.7, *p* < 0.0001; ANOVA: *F* = 50.8, *p* < 0.0001), indicating faster reaction times for the congruent stimuli (Mdn = 442.9, MAD = 30.18, *M* = 451.7, *SD* = 53.59) than for the incongruent ones (Mdn = 478.9, MAD = 40.8, *M* = 482.5, *SD* = 61.46), and thus a significant Stroop effect. Further, analyzes revealed a significant interaction between “Subject Gender” and “Stimulus Gender” (Rank test: *T* = 7.78, *p* = 0.0053 < alpha* = 0.0073; ANOVA: *F*_(1, 28)_ = 7.24, *p* = 0.0119). Male subjects showed a faster response to male than female stimuli, whereas female subjects responded faster to female than to male stimuli (Figure 2A, Table 1). Apart from the described results, no other comparisons reached significance.

**Table 3:** **Inference statistics for the reaction times**.

**RT**	**Rank test**			**Anova**		
	***t***	***df***	***p***	***F***	***df***	**p**
Subject G	0.449	1	0.5029	0.3790	1,28	0.5431
Stimulus G	0.491	1	0.4837	0.2812	1,28	0.6001
Congruency	34.683	1	<0.0001^*^	50.7859	1,28	0.0000
Subject G × Stimulus G	7.785	1	0.0053^*^	7.2386	1,28	0.0119
Subject G × Cong	2.539	1	0.1111	3.3514	1,28	0.0778
Stimulus G × Cong	0.696	1	0.4041	0.0136	1,28	0.9081
Subject G × Stimulus G × Cong	0.555	1	0.4562	0.0270	1,28	0.8707

Test results are shown for Brunner and Langer's (non-parametric) rank-based f1.LD.f2 test and the (parametric)repeated-measures ANOVA. Significant factors are indicated by an asterisk (^*^). Assessment whether a factor became significant or not, solely relies on the non-paramtric test. The adjusted apha for 7 tests is alpha^*^ = 1−(1−0.05)^1/7^ = 0.0073. G, Gender; Cong, Congruency.

### 3.3. Stroop effects

Stroop effects (Figure 2B and Table 4) varied between female and male subjects, with female subjects in general showing smaller Stroop effects (*M* = 16.8, MAD = 20.3, *M* = 22.9, *SD* = 36.7) than male subjects (Mdn = 29.8, MAD = 15.8, *M* = 38.7, *SD* = 28.9). However, this difference was only marginally significant (Rank Test: *t* = 5.254, *df* = 1, *p* = 0.0219 < 0.10^1/3^ = 0.034). No (not even marginally) significant differences were found for the Stroop effects toward female and male traffic light figures or the interaction (Figure 2B and Table 5).

**Table 4:** **Descriptive Statistics for the Stroop effect, i.e., reaction time differences between congruent and incongruent conditions**.

**Subject**	**Stimulus**	**Reaction times**					
**gender**	**gender**	**Mdn**	**MAD**	***M***	***SD***	***n***	***SE***
F	M	18.3	21.4	22.7	32.0	15	8.3
F	F	10.1	13.2	23.1	41.9	15	10.8
M	M	29.8	16.5	39.9	30.4	15	7.9
M	F	29.7	14.5	37.5	28.4	15	7.3
Both	M	20.7	19.4	31.3	31.9	30	5.8
Both	F	25.7	18.8	30.3	35.9	30	6.6
F	Both	16.8	20.3	22.9	36.7	30	6.7
M	Both	29.8	15.8	38.7	28.9	30	5.3

*Mdn, median; MAD, median absolute distance; M, mean; SD, standard deviation; number and SE, standard error*.

**Table 5:** **Inference statistics for the stroop effects**.

**Stroop**	**Rank test**			**Anova**		
	***t***	***df***	***p***	***F***	***df***	***p***
Subject G	5.254	1	0.0219	3.3514	1,28	0.0778
Stimulus G	0.145	1	0.7030	0.0136	1,28	0.9081
Subject G × Stimulus G	0.078	1	0.7806	0.0270	1,28	0.8707

Test results are shown for Brunner and Langer's (non-parametric) rank-based f1.LD.f1 test and the (parametric) repeated-measures ANOVA. Assessment whether a factor became significant or not, solely relies on the non-paramtric test. The adjusted alpha for 3 tests is alpha^*^ = 1−(1−0.05)^1/3^ = 0.01695. Only the factor “Subject Gender” became marginally significant. G, Gender.

## 4. Discussion

We investigated the accuracy (Acc), speed (RT), and robustness (Stroop effect) by which observers perceive female and male traffic light figures in congruent and incongruent stimulus conditions. We observed that subjects were faster in the congruent than in the incongruent conditions, which constitutes a Stroop effect. Generally, our results show that both, color and shape carry information in traffic signs. Regarding the type of figure we had found in a recent study that, while West and East German traffic light figures in their congruent form were equally effective in producing responses with high speed (RT) and high accuracy, the shape of the East German “Ampelmännchen” was more robust against conflicting information (smaller Stroop effect). Moreover, in the color task, (incongruent) East German figures were more distracting than West German ones. These findings likely result from the East traffic light figures being more visually “expressive” than their West German counterparts. In the present study we continued this line of investigation by testing how the recently introduced female traffic light figures compare to their male counterparts. By using both female and male subjects, we also investigated potential interactions between the gender of the traffic lights and the observers.

Our results showed that there was no main effect of the gender of the traffic light figures, neither for accuracy nor for reaction times. Also, the robustness of the figures as measured by the Stroop effect was similar – unlike for the West and East German figures tested in the previous study. This finding may suggest that no differences in performance are to be expected for the use of female vs. male Ampelmännchen signs in real-life traffic, where they guide both, female and male pedestrians.

The data revealed a significant interaction between the gender of the subjects and the gender of the figures on the traffic lights. Male subjects responded faster to male than to female traffic light figures, whereas female subjects responded faster to female than to male stimuli. Thus, the data are not in line with the other three hypotheses, which would have been associated with a faster response to (i) male figures (decade-long adaptation in the real world, or familiarity), (ii) female figures (novelty), or (iii) to figures of the opposite sex (attraction), respectively.

Of these refuted hypotheses, the explanation of familiarity (i) vs. novelty (ii) is interesting with regard to our previous study (Peschke et al., 2013). Comparing West to East German figures, we had found a higher degree of robustness and faster reaction times for the East German signs. We then had argued that this advantage results from the fact that the East German sign appears to be more expressive and the meaning of the figure more clearly apparent. Given that more subjects were familiar with the West than with the East German figure, we were able to rule out any familiarity effect in the sense of the familiarity/adaptation hypothesis (i). The fact that also the present data do not point into this direction increases our trust in this finding.

At the same time, our new data also rule out the opposite hypothesis (ii, novelty). Since our subjects were from West Germany, the East German signs appeared new to them, and the female sign even more. Thus, a novelty effect would have shown in a generally better performance for the female figure, which we did not find. This observation leads us to assume that neither familiarity nor novelty played any crucial role in the two studies, but that the effects found here can indeed be attributed to the different appearances of the figures.

In contrast to hypotheses (i) adaptation/familarity, (ii) novelty and (iii) attraction, the data obtained here strongly support the identification hypothesis (iv) that subjects react faster to figures of their own gender.

Our findings are thus in line with the social learning theory (Bandura, 1977), and the reported same-gender identification bias (e.g., Bandura et al., 1963; Slaby and Frey, 1975; Perloff, 1982; Gilbert et al., 1983; Bussey and Bandura, 1984; Killian, 1990). These experiments had shown that humans show a bias to attend to and learn from models of their own gender. This bias was irrespective of the age groups (children, adolescents, adults) and of the behavior type (aggressive, social). A recent neuroscientific study has additionally shown that observing same-sex models activates cortical areas regulating self-reward more strongly than observing opposite-sex models (Losin et al., 2012). The bias to attending same-sex models was also observed when the same-sex model fulfilled unpleasant duties (Perloff, 1982). The task used there is similar to our traffic light situation in that the model, that is the traffic light figure, fulfills the unpleasant duty of waiting at red light, while pedestrians are expected to follow that model. We thus conclude that the presented results may be explained by the same-gender bias in social learning. This connection may also explain our previous findings (Peschke et al., 2013). Bandura (1977) and Bussey and Bandura (1984) argue that the degree of learning depends on the degree to which the learner can identify with the model. Apparently, identifying with East German Ampelmann figures that are often described as looking “human-like” or even “cute” (see above) is easier than identifying with its West German counterparts that are more abstract.

Irrespective of the assumed theoretical background, our data show that introducing traffic lights with both (male and female) figures could help subjects to respond faster and possibly with a higher compliance due to the mechanisms of identification. Other studies in the field showed that personal factors such as the pedestrians' age, gender and group size are significantly correlated with the amount of jaywalking (for a recent literature review, see Brosseau et al., 2013), and recently successfully established positive effects of reduced waiting times (Brosseau et al., 2013), clear imperative signals (i.e., just red and green) (Stasi et al., 2014), and countdown displays (e.g., Lipovac et al., 2013), or proposed even camera-based adaptive green and red light times (Xiao et al., 2013). Complementing these approaches, we propose here to investigate whether the same-sex bias demonstrated by the Stroop effect really reflects a higher identification by testing in more realistic experiments whether pedestrians comply to a higher degree to traffic lights specific to their gender. We are aware that the introduction of female traffic lights as investigated here would address mostly the subgroup that jaywalks less frequently in comparison to males or teenagers (e.g., Lipovac et al., 2013). However, even male pedestrians might profit from introducing male and female figures on new traffic lights. Such lights might increase identification with the male lights by means of contrast to the female lights with which male pedestrians do not identify.

Another potential target of future research could be children. It may be interesting to learn whether children respond faster to traffic light figures depicting children, that is, peers to identify with, than figures that resemble adults such as the ones from whom they learn traffic rules. This way, the chance of identification would be higher and the signals might become more readily accepted.

To conclude, female and male traffic light figures appear to work similarly well when perceived by a mixed group of pedestrians. Optimization in terms of stronger identification with each gender group might result in even higher visual efficacy.

A central limitation of our study is the specific sample of young adults. In the light of the social psychological studies cited above, illustrating that the gender-gender interaction is a phenomenon that shows across all age groups, we hypothesize that the effect we describe can be generalized to other age groups. Certainly, this assumption needs to be tested with a wider range of subjects.

Apart from that, as novel signs, such as the female figures did not seem to confuse subjects or change their response characteristics significantly in a negative way, the most critical issue that opposes a widespread replacement of traffic light symbols may be the cost factor. However, with the advent of LED lights in computer-driven traffic light systems, the need to decide between male and female figures may also become obsolete, since figures could be exchanged easily through an update of the software.

## Author contributions

FK, BO, and CH conceived the experiments, FK conducted the experiments and analyzed the results. All authors wrote and reviewed the manuscript.

## Funding

Research was supported by a grant from the German Research Council (DFG) to BO (OL-297/9-1) and CH (HI-1286/3-1).

### Conflict of interest statement

The authors declare that the research was conducted in the absence of any commercial or financial relationships that could be construed as a potential conflict of interest.
